# Supplementary material for: Estimating the size distribution of plastics ingested by animals
Source: Nat Commun. 2020 Mar 27;11:1594. doi: 10.1038/s41467-020-15406-6 (PMC7101383; doi:10.1038/s41467-020-15406-6)
Supplement: Supplementary file 2 — Reporting Summary [file 41467_2020_15406_MOESM2_ESM.pdf]

nature research

Corresponding author(s): Prof Isabelle Durance

Last updated by author(s): March 4th 2020

Reporting Summary

Nature Research wishes to improve the reproducibility of the work that we publish. This form provides structure for consistency and transparency in reporting. For further information on Nature Research policies, see Authors & Referees and the Editorial Policy Checklist .

Statistics

For all statistical analyses, confirm that the following items are present in the figure legend, table legend, main text, or Methods section.

n/a	Confirmed	
☐	☒	The exact sample size (*n*) for each experimental group/condition, given as a discrete number and unit of measurement
☐	☒	A statement on whether measurements were taken from distinct samples or whether the same sample was measured repeatedly
☐	☒	The statistical test(s) used AND whether they are one- or two-sided<br>*Only common tests should be described solely by name; describe more complex techniques in the Methods section.*
☐	☒	A description of all covariates tested
☐	☒	A description of any assumptions or corrections, such as tests of normality and adjustment for multiple comparisons
☐	☒	A full description of the statistical parameters including central tendency (e.g. means) or other basic estimates (e.g. regression coefficient) AND variation (e.g. standard deviation) or associated estimates of uncertainty (e.g. confidence intervals)
☐	☒	For null hypothesis testing, the test statistic (e.g. *F*, *t*, *r*) with confidence intervals, effect sizes, degrees of freedom and *P* value noted<br>*Give P values as exact values whenever suitable.*
☒	☐	For Bayesian analysis, information on the choice of priors and Markov chain Monte Carlo settings
☒	☐	For hierarchical and complex designs, identification of the appropriate level for tests and full reporting of outcomes
☒	☐	Estimates of effect sizes (e.g. Cohen's *d*, Pearson's *r*), indicating how they were calculated

Our web collection on statistics for biologists contains articles on many of the points above.

Software and code

Policy information about availability of computer code

Data collection	On the 26th of January 2018 we used Web of Science (version 5.27) to find peer-reviewed research articles from 1900-2018 on plastic ingestion by any organism using the search string ((plastic OR plastics OR microplastic* OR mesoplastic* OR macroplastic*) AND (ingest* OR absorb* OR devour* OR eat* OR digest* OR consum* OR swallow* OR ingurgutat* OR engorg* OR gorge OR graz* OR masticat* OR ruminat* OR prey OR meal OR nourish* OR diet OR sustenance OR gastro* OR stomach* OR intest* OR assimili* OR incorporat* OR embod* OR engulf* OR envelop*) NOT (consumer)) under the heading “Topic”. We searched Science Citation Index Expanded (SCI-EXPANDED) --1900-present; Social Sciences Citation Index (SSCI) --1956-present; Arts & Humanities Citation Index (A&HCI) --1975-present; Conference Proceedings Citation Index- Science (CPCI-S) --1990-present; Conference Proceedings Citation Index- Social Science & Humanities (CPCI-SSH) --1990-present; Emerging Sources Citation Index (ESCI) --2015-present. Other software: ImageJ (version 1.51J8), ‘stats’ package (version 3.4.3), R (version 3.6.3; ‘Kite-Eating Tree’), ArcGIS (version 10.5.1).
Data analysis	Microsoft Excel version 16.16.7 and R (version 3.6.1; "Action of the toes"), within the RStudio environment (version 1.1.463).

For manuscripts utilizing custom algorithms or software that are central to the research but not yet described in published literature, software must be made available to editors/reviewers. We strongly encourage code deposition in a community repository (e.g. GitHub). See the Nature Research guidelines for submitting code & software for further information.

Data

Policy information about availability of data

All manuscripts must include a data availability statement. This statement should provide the following information, where applicable:

- Accession codes, unique identifiers, or web links for publicly available datasets
- A list of figures that have associated raw data
- A description of any restrictions on data availability

Raw data supporting the findings of this study are available as Supplementary Information. The 1,999 articles gathered on the 26th of January 2018, on Web of Science, listed by most relevant, with notes on the reasons for acceptance or rejection, are also included.

Field-specific reporting

Please select the one below that is the best fit for your research. If you are not sure, read the appropriate sections before making your selection.

☐ Life sciences ☐ Behavioural & social sciences ☒ Ecological, evolutionary & environmental sciences

For a reference copy of the document with all sections, see nature.com/documents/nr-reporting-summary-flat.pdf

Ecological, evolutionary & environmental sciences study design

All studies must disclose on these points even when the disclosure is negative.

Study description

Here, via systematic review of peer-reviewed primary research literature, we collect data on plastic ingestion by approximately 1,890 wild animals to generate an ecologically relevant, allometric relationship; validated on recently published data, to estimate the maximum size of plastic any animal may ingest, based on body length.

Research sample

Articles accepted for data collection reported or illustrated (e.g. via image analysis): (1) the size of the longest axis of ingested plastic (any plastic type) by a species of animal or a single animal; (2) the mean, mid-range or mean of size-bin mid-ranges for the body length of species or individuals containing ingested plastic. Data included as approximations of total body length were reported measurements of capitulum length, curved carapace length, and carapace width. The length of bivalves was recorded as the length of the shell. Descriptions of the exact dimensions measured of animal lengths were often unclear. Here we defined “total body length” as the distance from the most anterior to the most posterior part of an animal. Whether a measurement was classified as “total body length” usually required judgment of the methodology described.

Sampling strategy

Data collected from all research articles that met the inclusion criteria of the systematic review.

Data collection

Data collected by IBJ and FMW. Data stored in excel spreadsheet format. Data provided as CSV file.

Articles that provided plastic and animal size data for each specimen within a study were relatively sparse. More common were summary statistics for a group of individuals of the same species. We, therefore, prioritised the collection of data on animal species (i.e. groups of individuals). Where data were available for individual animals within a group as well as for the group as a whole, only data for the latter were retained to avoid pseudo-replication. Any data on individual animals were summarised for the lowest ranked taxonomic group possible.

Data on single or smaller groups of individuals of greater taxonomic resolution was prioritised over summarised data for higher taxonomic levels.

Where data were available only for subgroups where different plastic measurements were made (e.g. in surface area in some individuals or in lengths for others), we used only data expressed as linear length. Where data were available only for a single animal, summary statistics for a group were replaced with the actual values recorded for that individual. The number of animals in a group was recorded.

The largest piece of plastic ingested by a group of individuals is likely to be more representative of the true maximum for an entire population than a single individual. Since matching a specific individual to a specific plastic fragment was seldom possible for groups of individuals, we used the mean body length in relation to a plastic fragment ingested by any group member. We focused on the precision of the relationship between body size and plastic size, by giving precedence to body length measurements of specimens that contained plastics (i.e. not all animals in a study would contain plastics), over data for wider groupings of animals (e.g. the mean body length of all animals in a study).

Ingested plastics were defined as those found in the main digestive tract of an animal via necropsy or tissue digestion. We excluded data on plastics in faeces or regurgitates, live animals or observations of plastic ingestion in behavioural studies. Regurgitated material might reflect material that could not be ingested further into the gastrointestinal tract while faeces would contain only those plastic fragments that could pass through the gastrointestinal tract and not be retained. Where available, the longest axis of the smallest ingested plastic fragment and the type of magnification used to detect plastics, was noted for each record. However, the absence of this information did not disqualify an article. The full list of raw data is available as Supplementary Material.

The use of reported values was prioritised, but in their absence, data were also collected on animal and plastic lengths from images using ImageJ (version 1.51J8). Measurements were made of the longest straight axis of a plastic fragment, calibrated according to the scale indicators in images, using a segmented line to measure long, coiled pieces of plastic material. Where coils of plastic could not be discriminated as a single piece, the maximum axis of the coil as a whole was measured. We used the image of highest resolution available. Only plastics that were wholly visible in an image were measured.

Decimal degree latitude and longitude coordinates were approximated from reported coordinates, or site descriptions where coordinates were unavailable, using Latlong. In the case of many sampling sites, an approximate central point was used for all sites in a study.

The final data set contained 44 records, 41 of which were for individual species, the other three for multiple species contained within the Actinopterygii class. For the 41 species-level records, species were classified as “marine only”, “marine and brackish”, “marine, brackish and freshwater” or “freshwater only”, according to FishBase27 and SeaLifeBase28. Any records from SeaLifeBase were classified as “marine only” by default, with additional descriptions of tendencies for brackish or freshwater environments added to fit one of the four water type classifications. For 34 of these 41 species, data on depth range were available on either FishBase or SeaLifeBase.

Timing and spatial scale

On the 26th of January 2018 we used Web of Science (version 5.27) to find peer-reviewed research articles from 1900-2018 .

Data exclusions

The 22,205 records found were listed using the “Relevance” function within Web of Science and the 1,999 most relevant results were exported as a .txt file for screening. All 1,999 titles were screened for relevance (title screening), and abstracts were also read in cases where the reviewer was unsure. In all cases where the reviewer was unsure, the article was retained for further screening at

	the next phase (full text screening). To be considered for the Data collection phase (below), an article was required to meet the following criteria: (1) Article seemed like it included some information on ingestion of any type or size of plastic by an organism; (2) article must report on field-based studies where plastics were present in the environment at natural concentrations and size distributions, as lab studies are often non-representative in terms of plastic availability. We excluded reports of plastic consumption by humans and reviews. Only peer-reviewed primary research articles were accepted. These inclusion criteria were pre-established at the study design phase, and any records found not to meet these criteria were excluded, because they did not serve the purposes of the meta-analysis.
Reproducibility	See "Validation of the allometric relationship".
Randomization	Articles that provided plastic and animal size data for each specimen within a study were relatively sparse. More common were summary statistics for a group of individuals of the same species. We, therefore, prioritised the collection of data on animal species (i.e. groups of individuals). Where data were available for individual animals within a group as well as for the group as a whole, only data for the latter were retained to avoid pseudo-replication. Any data on individual animals were summarised for the lowest ranked taxonomic group possible.<br>Data on single or smaller groups of individuals of greater taxonomic resolution was prioritised over summarised data for higher taxonomic levels.<br>Where data were available only for subgroups where different plastic measurements were made (e.g. in surface area in some individuals or in lengths for others), we used only data expressed as linear length. Where data were available only for a single animal, summary statistics for a group were replaced with the actual values recorded for that individual. The number of animals in a group was recorded.<br>The largest piece of plastic ingested by a group of individuals is likely to be more representative of the true maximum for an entire population than a single individual. Since matching a specific individual to a specific plastic fragment was seldom possible for groups of individuals, we used the mean body length in relation to a plastic fragment ingested by any group member. We focused on the precision of the relationship between body size and plastic size, by giving precedence to body length measurements of specimens that contained plastics (i.e. not all animals in a study would contain plastics), over data for wider groupings of animals (e.g. the mean body length of all animals in a study). This meta-analysis is wholly dependent on literature published at the data collection phase, and as such, reproducibility testing was not appropriate. We draw attention to "Validation of the allometirc realtionship" as a rough surrogate, where we tested the allometric realonship by randomly sampling validaton and parameterisation data.
Blinding	Blinding not employed.

Did the study involve field work? ☐ Yes ☒ No

Reporting for specific materials, systems and methods

We require information from authors about some types of materials, experimental systems and methods used in many studies. Here, indicate whether each material, system or method listed is relevant to your study. If you are not sure if a list item applies to your research, read the appropriate section before selecting a response.

Materials & experimental systems

n/a	Involved in the study
☒	☐ Antibodies
☒	☐ Eukaryotic cell lines
☒	☐ Palaeontology
☒	☐ Animals and other organisms
☒	☐ Human research participants
☒	☐ Clinical data

Methods

n/a	Involved in the study
☒	☐ ChIP-seq
☒	☐ Flow cytometry
☒	☐ MRI-based neuroimaging
